# Pulse Pressure: An Emerging Therapeutic Target for Dementia

**DOI:** 10.3389/fnins.2020.00669

**Published:** 2020-06-24

**Authors:** Rachel A. Levin, Mark H. Carnegie, David S. Celermajer

**Affiliations:** ^1^The Brain Protection Company, Sydney, NSW, Australia; ^2^M.H. Carnegie & Co., Sydney, NSW, Australia; ^3^The Heart Research Institute, Sydney, NSW, Australia

**Keywords:** pulse pressure, carotid wave intensity, blood brain barrier, inflammation, oxidation, amyloidogenesis, microbleed, cognitive decline and dementia

## Abstract

Elevated pulse pressure can cause blood-brain barrier dysfunction and subsequent adverse neurological changes that may drive or contribute to the development of dementia with age. In short, elevated pulse pressure dysregulates cerebral endothelial cells and increases cellular production of oxidative and inflammatory molecules. The resulting cerebral microvascular damage, along with excessive pulsatile mechanical force, can induce breakdown of the blood-brain barrier, which in turn triggers brain cell impairment and death. We speculate that elevated pulse pressure may also reduce the efficacy of other therapeutic strategies for dementia. For instance, BACE1 inhibitors and anti-amyloid-β biologics reduce amyloid-β deposits in the brain that are thought to be a cause of Alzheimer’s disease, the most prevalent form of dementia. However, upregulation of oxidative and inflammatory molecules and increased amyloid-β secretion by cerebral endothelial cells exposed to elevated pulse pressure may hinder cognitive improvements with these drugs. Additionally, stem or progenitor cell therapy has the potential to repair blood-brain barrier damage, but chronic oxidative and inflammatory stress due to elevated pulse pressure can inhibit stem and progenitor cell regeneration. Finally, we discuss current efforts to repurpose blood pressure medications to prevent or treat dementia. We propose that new drugs or devices should be developed to safely reduce elevated pulse pressure specifically to the brain. Such novel technologies may alleviate an entire downstream pathway of cellular dysfunction, oxidation, inflammation, and amyloidogenesis, thereby preventing pulse-pressure-induced cognitive decline. Furthermore, these technologies may also enhance efficacy of other dementia therapeutics when used in combination.

## Introduction

Force from ventricular ejection produces a pulse pressure in the arterial tree. Pulse pressure (systolic minus diastolic blood pressure) is normally dampened by the elastic properties of central arteries. However, pulse pressure can become elevated in some circumstances, especially with increased age (Pinto, 2007). Two mechanisms that lead to a chronic increase in pulse pressure are (i) progressive stiffening of central vessels from changes in endothelial cell and vessel wall structure (Lee and Oh, 2010; Wagenseil and Mecham, 2012) and (ii) excessive wave reflection from high resistance peripheral vessels (Safar, 2008). Age-related elevation of pulse pressure is typically due to increased systolic pressure, while diastolic pressure is unchanged or slightly decreased (Pinto, 2007; Steppan et al., 2011). High systolic pressure increases the workload on the left ventricle, which can result in left ventricular hypertrophy and heart failure (Chae et al., 1999; Steppan et al., 2011). The kidneys and brain are also adversely affected by arterial stiffening and high pulse pressure, as these organs share the key characteristic of having low resistance microvasculature that allows for deep penetration of the pulse (O’Rourke and Safar, 2005). Thus, both organs are susceptible to damage of their delicate microvessels, which in turn damages the organ tissue (Arulkumaran et al., 2010; Stone et al., 2015). Elevated pulse pressure can therefore lead to comorbidities such as chronic kidney disease and cognitive decline (Townsend, 2015). In this perspective article, we will focus on the impact of high pulse pressure on the brain.

Throughout the human brain, over 600 kilometers of blood vessels supply the brain tissue with oxygen and nutrients, while also removing metabolic by-products from the brain (Zlokovic, 2008; Pardridge, 2015; Iadecola, 2017; Kisler et al., 2017). Cerebral microvessels lack external elastic laminae, making them more fragile than other systemic vessels (Lee, 1995). Neuronal health and signaling rely on precise chemical homeostasis; hence, blood vessels in the brain are specialized to form the blood-brain barrier, a structure that restricts non-selective passage of molecules and cells. The blood-brain barrier is composed of endothelial cells connected by tight junctions and pericytes that encircle the microvessels, which together are sheathed by astrocyte end feet (Ballabh et al., 2004). Tight junctions block the leakage of blood-based molecules in between individual cerebral endothelial cells; pericytes regulate endothelial cell gene expression and polarize astrocyte end feet; and astrocytes secrete factors that support the development and maintenance of cellular interactions within the blood-brain barrier and between the blood-brain barrier and neurons (Armulik et al., 2010; Alvarez et al., 2013). Breakdown of the blood-brain barrier is widely believed to drive several neurodegenerative diseases such as Parkinson’s disease, Huntington’s disease, and dementia, including Alzheimer’s disease (Ballabh et al., 2004; Desai et al., 2007; Drouin-Ouellet et al., 2015; Wardlaw et al., 2017; Sweeney et al., 2018).

Development of dementia with age is likely driven by numerous distinct and multifactorial pathologies. As the discovery and understanding of underlying mechanisms advances, we expect new sub-types of dementia to be defined. This may enable development of therapeutic strategies that are efficacious for specific patient groups. A rapidly growing body of research studies and epidemiologic evidence indicate that elevated pulse pressure is a potential key contributor to blood-brain barrier breakdown and cognitive impairment in many individuals (Stone et al., 2015; Thorin-Trescases et al., 2018). High pulse pressure correlates with cerebral microvascular damage (Triantafyllidi et al., 2009) as well as white matter structural differences in elderly patient brains (Tsao et al., 2013; Purkayastha et al., 2014; Tarumi et al., 2014). In multiple large population studies considering thousands of individuals, high pulse pressure has been a strong, independent risk factor and predictor of cognitive decline later in life (Waldstein et al., 2008; Mitchell et al., 2011; Meyer et al., 2017). Most recently, Chiesa et al. (2019) linked progressive cognitive impairment to carotid wave intensity, a surrogate marker of arterial stiffness and pulse pressure directly delivered to the brain. Middle-aged adults with top-quartile carotid wave intensity values were found to have a 50% increased risked of future cognitive decline, compared to those with “control value” carotid wave intensity. Further, de Montgolfier et al. (2019) demonstrated that high pulse pressure in wild-type mice and Alzheimer’s disease model mice increased the prevalence of microbleeds, which is characteristic of individuals with Alzheimer’s disease (Brundel et al., 2012). Taken together, these findings highlight pulse pressure as a new pathogenetic factor for cognitive decline.

There are nearly 50 million people living with dementia worldwide, and this number is expected to triple by 2050 (World Health Organization, 2017). Therapeutic development efforts to date have largely focused on directly reducing amyloid-β, a marker of Alzheimer’s disease, in the brain. Yet, despite billions of dollars spent on R&D, no cure or preventative solution has earned FDA approval, emphasizing the urgent need for novel approaches. Here, we discuss the impacts of elevated pulse pressure on the blood-brain barrier and cognition, and we propose that pulse pressure is a promising therapeutic target for a potential new sub-type of dementia.

## Pulse Pressure, Endothelial Cell Dysfunction, and blood-brain Barrier Damage

Endothelial cell dysfunction is thought to be critically involved in development and progression of several diseases such as atherosclerosis, heart failure, kidney disease, and certain neurological conditions (Su et al., 2006; Malyszko, 2010; Rajendran et al., 2013; Vanhoutte et al., 2017; Giannitsi et al., 2019). Pulse pressure regulates endothelial cells in diverse ways, ranging from the individual cell level to the greater microvascular integrity level. Elevated pulse pressure (>70 mmHg) causes blood vessels to be cyclically stretched ∼15–20% (pathological stretch) compared to normal pulse pressure (30–50 mmHg) that causes blood vessels to be cyclically stretched only ∼5% (physiological stretch) (Anwar et al., 2012; Gao et al., 2014; Jufri et al., 2015). Physiological stretch is essential for maintaining proper endothelial cell gene expression and function such as signal transduction, balanced reactive oxygen species generation and cellular structure. Conversely, pathological stretch has been shown to cause oxidative stress, inflammation, and apoptosis of endothelial cells (Jufri et al., 2015) that could compromise the blood-brain barrier.

Pathological stretch increases production of reactive oxygen species and inflammatory cytokines by endothelial cells. Increased O${}_{2}^{-}$ promotes oxidative tissue damage and increases H_2_O_2_ that activates the NF-κB inflammatory pathway. Inflammatory cytokines, including VCAM-1, ICAM-1, TNFα, IL-6, and IL-8, further activate NF-κB and inflammation in blood vessels (Jufri et al., 2015). Blood concentrations of VCAM-1, TNFα, and IL-6 are higher in people with Alzheimer’s disease compared to healthy individuals (Swardfager et al., 2010; Lai et al., 2017). Chronic inflammation of the blood-brain barrier can lead to apoptosis of cerebral endothelial cells, astrocytes, and pericytes (van Kralingen et al., 2013; Jufri et al., 2017; Sweeney et al., 2018). Loss of these cells may permanently impair blood-brain barrier integrity since NF-κB activation, chronic inflammation, and oxidative stress also cause stem/progenitor cell dysfunction (Yao et al., 2006; Shao et al., 2011; Lin et al., 2013; Josephson et al., 2019) that could diminish regenerative potential in the blood-brain barrier. Additionally, the NF-κB pathway upregulates amyloidogenesis (Ju Hwang et al., 2019). Upregulated β-secretase 1 (BACE1) and amyloid precursor protein (APP) expression and increased amyloid-β (specifically Aβ42) secretion have all been directly observed from cerebral endothelial cells in response to pathological stretch (Gangoda et al., 2018). Amyloid-β decreases tight junction proteins, increases IL-6, and increases matrix metalloproteinases that degrade the extracellular matrix (Vukic et al., 2009; Hartz et al., 2012; Weekman and Wilcock, 2016). TNFα also upregulates matrix metalloproteinase expression in endothelial cells under pathological stretch (Wang et al., 2003). Thus, pathological stretch modulates numerous molecules that result in chronic oxidative stress, inflammation, amyloidogenesis, and damage of the blood-brain barrier.

Pathological stretch can also impact blood-brain barrier integrity through non-oxidative and non-inflammatory pathways. Pathological stretch upregulates integrin β3 and downregulates titin in cerebral endothelial cells, which may reduce cellular elasticity, consequently damaging the blood-brain barrier. Furthermore, downregulation of eukaryotic translation initiation factor 4 gamma 3 in these cerebral endothelial cells under excess stretch attenuates global protein synthesis and therefore cell proliferation (Jufri et al., 2017). Sorting nexin-1, a protein that recycles cell-surface receptors (Haft et al., 1998), is also substantially downregulated in cerebral endothelial cells exposed to pathological stretch (Jufri et al., 2017). While the specific interaction between sorting nexin-1 and cerebral endothelial cell receptors is not yet defined, dysregulation of sorting nexins often results in abnormal receptor expression and cellular signaling that disrupts homeostasis (Zhao et al., 2012; Wang et al., 2013; Yang et al., 2014). Thus, decreased sorting nexin-1 due to pathological stretch has the potential to dysregulate key endothelial cell receptors that may exacerbate microvascular damage. For example, sorting nexin downregulation decreases expression of the endothelial cell surface receptor FEEL-1/stabilin-1 (Adachi and Tsujimoto, 2010), which can reduce endothelial cell-cell interaction and angiogenesis (Adachi and Tsujimoto, 2002).

blood-brain barrier breakdown due to elevated pulse pressure may result in microbleeds in the brain from cumulative pulse-pressure-induced cellular damage over time as well as from the excessive direct mechanical force of the pulse. As briefly mentioned earlier, a recent mechanistic study in wild-type mice and Alzheimer’s disease model (APP/PS1) mice has reinforced the importance of high pulse pressure in the pathogenesis of dementia-related cerebral changes. de Montgolfier et al. (2019) studied mice following transverse aortic constriction surgery that increased pulse pressure in only the right side of the brain, while the left side of the brain experienced normal pulse pressure and served as an internal control. In addition to causing more microbleeds, elevated pulse pressure led to blood-brain barrier dysfunction, loss of cerebral microvessel density, and hypoperfusion in both wild-type and APP/PS1 mice. Notably, the right hemisphere that was exposed to high pulse pressure in APP/PS1 mice also had more amyloid-β deposition compared to the left hemisphere that experienced normal pulse pressure.

## blood-brain Barrier Damage and Cognitive Decline

**FIGURE 1:**
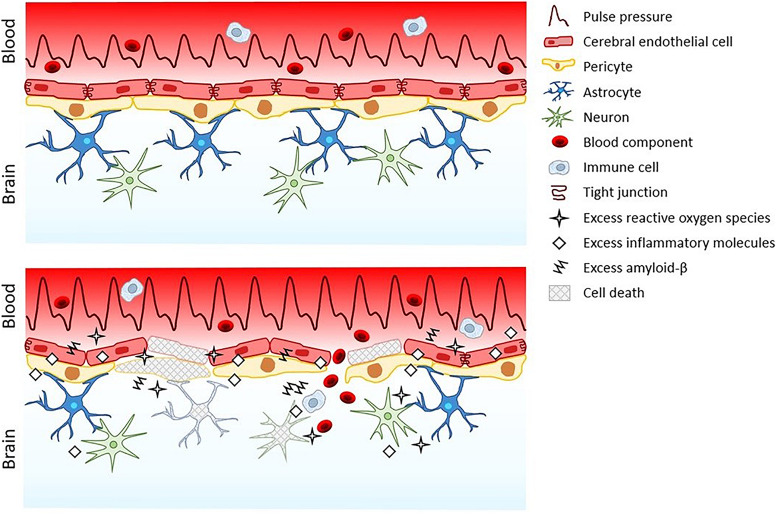
The blood-brain barrier and neuropil under normal pulse pressure (top) versus elevated pulse pressure (bottom).

Elevated pulse pressure compromises the blood-brain barrier through several processes, including oxidation, inflammation, and apoptosis. However, this damage does not stay confined to the blood-brain barrier (Figure 1). Reactive oxygen species, inflammatory cytokines, amyloid-β, and blood leak into the neural tissue from the injured blood-brain barrier triggering neuron dysfunction and death that may drive the development of certain dementias (Lynch, 2010; Sharma and Sharma, 2010; Van der Flier and Cordonnier, 2012; Lyman et al., 2014; Martinez-Ramirez et al., 2014; Huang et al., 2016; Sweeney et al., 2018). Resultant oxidative stress and inflammation in the brain following blood-brain barrier leakage can also upregulate brain cell production of amyloid-β (Tong et al., 2005; Lee et al., 2008). This increase in amyloid-β, coupled with the increased amyloid-β secretion from cerebral endothelial cells exposed to elevated pulsatile stretch (Gangoda et al., 2018), accelerates the formation of amyloid-β plaques (Alasmari et al., 2018; Cheignon et al., 2018). Amyloid-β deposition in the brain disrupts neuronal synapses and breaks neuronal branches (Tsai et al., 2004) and further exacerbates oxidative stress, neuroinflammation, and apoptosis (Miranda et al., 2000; Xie et al., 2013; Dorey et al., 2014). Moreover, loss of pericytes due to blood-brain barrier inflammation contributes amyloid-β build-up since pericytes can remove amyloid-β from the brain (Sagare et al., 2013).

blood-brain barrier microbleeds from elevated pulse pressure allow for systemic molecules and circulating cells to enter the sensitive neural tissue. For example, hemoglobin is a vital systemic protein that carries oxygen in blood, but it can damage neural tissue. Breakdown of hemoglobin in the brain leads to release of redox-active iron and production of reactive oxygen species resulting in oxidative damage (Robinson et al., 2009). Heme-deposits colocalize with amyloid-β plaques, indicating that microbleeds are involved in amyloid-β pathology (Cullen et al., 2006). Another important systemic protein is fibrinogen, which is a clotting factor in blood. However, upon vascular injury, fibrinogen is enzymatically converted to fibrin, which can also damage neural tissue. Brain deposition of fibrinogen originating from blood is increased in Alzheimer’s disease patients (Narayan et al., 2015). The resulting fibrin is linked to neuroinflammation, neuron dysfunction, and neuron death, as well as reduced blood-brain barrier integrity (Paul et al., 2007; Cortes-Canteli et al., 2015). Fibrin can bind to amyloid-β, which prevents fibrin clearance from both the blood-brain barrier and the brain leading to more microbleeds and neurodegeneration (Cortes-Canteli et al., 2012; Ahn et al., 2017). Microbleeds also recruit circulating immune cells that infiltrate the brain (Fiala et al., 2002; Cullen et al., 2005) and activate the brain-resident microglia (Xue and Del Bigio, 2000), which in turn activate neurotoxic reactive astrocytes that are implicated in neurodegeneration (Liddelow et al., 2017). However, even in the absence of microbleeds, pulse-pressure-induced endothelial dysfunction alone may be sufficient to drive wide-spread degeneration of the blood-brain barrier and neural tissue.

**FIGURE 2:**
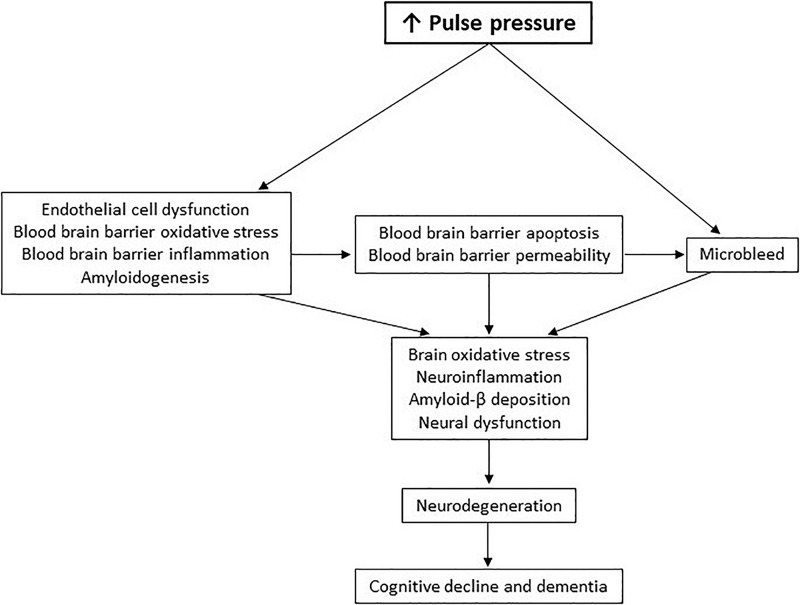
The pulse-pressure-induced cognitive decline pathway.

In summary, elevated pulse pressure delivers initial and continuous insults to the blood-brain barrier. Chronic oxidation and inflammation in the blood-brain barrier and upregulated secretion of amyloid-β from the blood-brain barrier causes persistent brain oxidative stress, neuroinflammation, amyloid-β deposition, and consequential neurodegeneration. This new pathological pathway of pulse-pressure-induced cognitive decline in dementia (Figure 2) may shed light on previous disappointments in therapeutic development for dementia, as well as reveal future opportunities.

## Potential Pulse Pressure Impacts on Therapeutic Strategies for Dementia

Amyloid-β has been a primary focus of R&D for Alzheimer’s disease. Despite over two decades of work and numerous clinical-stage drug candidates, no BACE1 inhibitors or anti-amyloid-β therapeutics have been demonstrated to improve cognitive function, thus none have achieved regulatory approval (Panza et al., 2019). Considering the pulse pressure paradigm of dementia (Figures 1, 2), drug targeting of only amyloid-β might be insufficient as a stand-alone therapy. Alternatively, anti-inflammatory agents have been proposed to treat Alzheimer’s disease. Anti-inflammatory agents like enetercept (a TNFα inhibitor approved for several types of arthritis) have been suggested to treat Alzheimer’s disease (Decourt et al., 2017). However, a phase II trial of enterecept failed to show any significant improvements in cognitive ability (Butchart et al., 2015). Other anti-inflammatory agents currently in clinical trials for Alzheimer’s disease include a RIPK1 inhibitor (DNL747, phase I; Denali Therapeutics), a TREM2 activator (AL002, phase I; Alector), and a non-steroidal anti-inflammatory (Salsalate, phase I; University of California San Francisco). Ultimately, targeting inflammatory-mediating molecules or amyloid-β to treat dementia may be an uphill battle since elevated pulse pressure will continue to broadly activate various inflammatory processes and increase amyloid-β throughout the blood-brain barrier and neural tissue. Thus, lowering elevated pulse pressure in certain dementia patients may be an essential first step to limit the production of inflammatory molecules and amyloid-β (as well as reactive oxygen species) before administering drugs, if necessary, to inhibit any remaining disease activity. Reduction of pulse pressure may therefore also allow for lower doses of these drugs to improve their safety profiles.

Another challenge for many neurological drug candidates, including anti-inflammatory and anti-amyloid-β biologics, is the inability to readily cross blood-brain barrier; only molecules that have a molecular weight under 400 Da and form fewer than eight hydrogen bonds are expected to passively diffuse (Pardridge, 2012). Hence, blood-brain barrier damage has been hypothesized as an avenue for larger drugs to enter the brain. Abulrob et al. (2008) showed that biologics can pass through blood-brain barrier lesions but do not widely distribute in neural tissue. Also, leakage is not uniform across the entire blood-brain barrier (Bien-Ly et al., 2015; Gyanwali et al., 2019), so drug delivery throughout the brain may be inadequate. Moreover, while blood-brain barrier disruption from elevated pulse pressure may enable some locally confined penetration of certain dementia drugs into the brain, it also allows for entry of toxic blood components that harm neurons. Hence, promoting blood-brain barrier health and integrity through the reduction of elevated pulse pressure alone may be a superior solution for treating certain dementias, and only if still required, drug candidates that can passively diffuse through the intact blood-brain barrier could be trialed.

A different drug delivery approach utilizes endogenous cerebral endothelial cell receptors that innately transport specific molecules from the systemic circulation into the brain. One such receptor is the insulin receptor that transports insulin from blood into the brain to support neuron function and metabolism (Bilotta et al., 2017). Molecular trojan horse technology is being developed to exploit various transport receptors. Molecular trojan horses are comprised of a therapeutic domain (e.g., an enzyme, an antibody fragment, or neurotrophin) fused to a receptor-binding domain (e.g., an insulin receptor antibody) to facilitate active transport by receptors across the blood-brain barrier (Pardridge, 2017). However, elevated pulse pressure causes endothelial cell dysfunction (Jufri et al., 2015; Jufri et al., 2017), which could impact receptor expression and activity. Therefore, it may be critical to first resolve elevated pulse pressure before administering therapies that have been designed in consideration of healthy blood-brain barrier dynamics.

Stem and progenitor cell therapies are also gaining attention as a strategy to repair blood-brain barrier damage and treat dementia (Cheng et al., 2018; Zhang et al., 2018; Alipour et al., 2019). Autologous and allogeneic stem cells are currently in clinical trials for Alzheimer’s disease (Hope Biosciences; Nature Cell Co; Medipost Co; CHA Biotech Co; Stemedica Cell Technologies; Longeveron; and University of Miami). However, elevated pulse pressure chronically induces NF-κB, inflammation, and reactive oxygen species production that can limit the regenerative potential of stem and progenitor cells (Yao et al., 2006; Shao et al., 2011; Lin et al., 2013; Josephson et al., 2019). Accordingly, reduction of elevated pulse pressure may be necessary to enhance stem and progenitor cell efficacy.

## Pulse Pressure as a Therapeutic Target for Dementia

As discussed above, elevated pulse pressure may initiate a cascade of oxidation, inflammation, amyloidogenesis, blood-brain barrier damage, and neurodegeneration. Therefore, novel therapeutics could be developed to target pulse pressure as a potential preventative solution or treatment for certain dementias. A relevant approach currently under investigation is the repurposing of blood pressure medications. In the SPRINT-MIND study of 9361 individuals, aggressive antihypertensive therapy was shown to significantly reduce the risk of cognitive decline (Williamson et al., 2019) but was also linked to serious adverse events, such as hypotension and acute kidney failure (Group, 2015). Currently, Losartan and Telmisartan are in phase III (University of Texas Southwestern) and phase I (Emory University) trials for Alzheimer’s disease, respectively. Both are angiotensin II receptor antagonists that induce blood vessel dilation to lower blood pressure, which may reduce the incidence, progression, and pathology of Alzheimer’s disease (Li et al., 2010, 2012). However, Losartan, Telmisartan, and the antihypertensive drugs in the SRPINT-MIND study lower both systolic and diastolic blood pressure. Lowering diastolic blood pressure is potentially dangerous in dementia patients if cerebral autoregulation fails in the elderly (Toth et al., 2017; Zhou et al., 2019) since this may result in decreased blood flow, which can exacerbate cognitive decline (Benedictus et al., 2017; Leijenaar et al., 2017). Considering the safety profiles and effect on diastolic blood pressure, these drugs may be difficult to apply in practice for treating older patients with stiff vessels.

When developing new technologies to restore healthier pulse pressure, minimizing adverse events will be paramount due to the elderly patient population in dementia. Accordingly, an appealing therapy would lower systolic but not diastolic blood pressure. If feasible, therapeutic targeting of the brain, instead of the whole systemic circulation, may further benefit safety. For example, reducing pulse wave intensity at and distal to the carotid artery may be particularly useful for alleviating pulse pressure impacts on the brain specifically. A technology that safely lowers elevated pulse pressure could also improve efficacy of potentially synergistic therapies when used in combination. Future therapeutic development could explore reducing carotid/cerebral artery stiffness, restoring carotid/cerebral artery elasticity, or reducing peripheral wave reflection. These approaches may produce a novel drug or device to prevent or treat cognitive decline in certain dementias.

## Author Contributions

The authors conceived the scope of the manuscript together. RL drafted the manuscript. All authors critically revised the manuscript. RL finalized the manuscript for publication. All authors approved the submitted version.

## Conflict of Interest

The Brain Protection Company is a clinical-stage company developing novel therapies for cognitive decline. DC is the Founder and Chief Medical Officer of The Brain Protection Company and holds equity in The Brain Protection Company. RL and MC are from M.H. Carnegie & Co, which holds equity in The Brain Protection Company.
